# High-Throughput Tissue Bioenergetics Analysis Reveals Identical Metabolic Allometric Scaling for Teleost Hearts and Whole Organisms

**DOI:** 10.1371/journal.pone.0137710

**Published:** 2015-09-14

**Authors:** Nishad Jayasundara, Jordan S. Kozal, Mariah C. Arnold, Sherine S. L. Chan, Richard T. Di Giulio

**Affiliations:** 1 Nicholas School of the Environment, Duke University, Durham, North Carolina, United States of America; 2 Medical University of South Carolina, Charleston, South Carolina, United States of America; University of Alabama at Birmingham, UNITED STATES

## Abstract

Organismal metabolic rate, a fundamental metric in biology, demonstrates an allometric scaling relationship with body size. Fractal-like vascular distribution networks of biological systems are proposed to underlie metabolic rate allometric scaling laws from individual organisms to cells, mitochondria, and enzymes. Tissue-specific metabolic scaling is notably absent from this paradigm. In the current study, metabolic scaling relationships of hearts and brains with body size were examined by improving on a high-throughput whole-organ oxygen consumption rate (OCR) analysis method in five biomedically and environmentally relevant teleost model species. Tissue-specific metabolic scaling was compared with organismal routine metabolism (RMO_2_), which was measured using whole organismal respirometry. Basal heart OCR and organismal RMO_2_ scaled identically with body mass in a species-specific fashion across all five species tested. However, organismal maximum metabolic rates (MMO_2_) and pharmacologically-induced maximum cardiac metabolic rates in zebrafish *Danio rerio* did not show a similar relationship with body mass. Brain metabolic rates did not scale with body size. The identical allometric scaling of heart and organismal metabolic rates with body size suggests that hearts, the power generator of an organism’s vascular distribution network, might be crucial in determining teleost metabolic rate scaling under routine conditions. Furthermore, these findings indicate the possibility of measuring heart OCR utilizing the high-throughput approach presented here as a proxy for organismal metabolic rate—a useful metric in characterizing organismal fitness. In addition to heart and brain OCR, the current approach was also used to measure whole liver OCR, partition cardiac mitochondrial bioenergetic parameters using pharmacological agents, and estimate heart and brain glycolytic rates. This high-throughput whole-organ bioenergetic analysis method has important applications in toxicology, evolutionary physiology, and biomedical sciences, particularly in the context of investigating pathogenesis of mitochondrial diseases.

## Introduction

The metabolic rate, often characterized as the rate of oxygen consumption, is a fundamental metric in physiological, ecological and evolutionary analyses of organismal survival and fitness. The metabolic rate of an organism demonstrates an allometric scaling relationship with body mass according to the equation *Y = a M* ^*b*^, where *Y* is metabolic rate, *M* is body mass, *a* is the species-specific scaling constant, and *b* is the scaling exponent [1–4]. The metabolic theory of ecology describes the biological significance of this relationship and provides a framework to predict how allometric scaling of metabolic rate versus body mass governs ecological processes at all levels of organization from individual organisms to the biosphere [5]. The scaling exponent *b* recently has received widespread attention with West and colleagues [6–8] suggesting that fractal-like vascular distribution networks of biological systems underlie the allometric scaling laws for individual organisms, single cells, intact mitochondria, and enzyme molecules [9].

However, tissue-specific metabolic rate scaling relationships and their correlations with whole organismal metabolic rates are notably absent from this paradigm. In the current study, we improved on a method developed by Little and Seebacher [10] to examine *ex vivo* heart-specific oxygen consumption rate (OCR) using the XF^e^24 Extracellular Flux Analyzer (Seahorse Bioscience, Billerica, MA) to explore (i) metabolic rate scaling of heart and brain tissues with organ size and body size and (ii) how these scaling relationships compare with whole organismal metabolic rates in five biomedically and environmentally relevant teleost model species—zebrafish (*Danio rerio*), Atlantic killifish (*Fundulus heteroclitus*), mosquito fish (*Gambusia holbrooki*), Japanese medaka (*Oryzias latipes*), and fathead minnow (*Pimephales promelas*). To investigate the metabolic scaling relationship between basal tissue-specific OCR and routine whole organismal metabolic rate (RMO_2_), we quantified whole organismal respiration rates for each fish prior to heart and brain measurements in all five species.

In addition to quantifying basal OCR for tissues, the XF^e^24 Extracellular Flux Analyzer can be used to obtain indices of tissue-specific mitochondrial bioenergetics with judicious use of different pharmacological agents [10,11]. Interest in mitochondrial function assessments is increasing in many contexts, ranging from biomedical sciences and toxicology to evolutionary and ecological physiology, as mitochondria play an essential role in aerobic ATP production and are involved in a number of other cellular processes [12–14]. Changes in mitochondrial structure and function are associated with a number of metabolic diseases and other disorders [15] and have been studied extensively. A key limitation in mitochondrial function analysis is that it is often assessed *in vitro* with isolated mitochondria or cells. While these approaches provide important insights into mitochondrial function, the results are likely to be confounded by perturbed cellular conditions [16]. Furthermore, these methods are often cumbersome and low throughput.

In the current study, to overcome aforementioned limitations, we attempted to partition certain components of mitochondrial function *ex vivo* based on whole tissue OCR measurements. We successfully used different pharmacological agents to obtain indices of OCR due to total mitochondrial and non-mitochondrial respiration, maximal respiration, and reserve mitochondrial capacity in heart tissues for two of the species (*D*. *rerio* and *F*. *heteroclitus*). While we were able to measure basal OCR for brain tissues in all five species and liver OCR in *D*. *rerio* and *F*. *heteroclitus*, our attempts to alter mitochondrial function in these tissues with pharmacological agents were less successful than for heart tissues. Furthermore, we also present a method for quantification of extra cellular acidification rate (ECAR; an index for glycolysis) for hearts and brains from juvenile and adult *D*. *rerio*.

Overall, the methods we describe here provide a rapid and relatively simple high-throughput approach to estimate mitochondrial bioenergetics *ex vivo* with intact hearts of small (~ <1g) teleost species and enable us to examine heart and brain metabolic scaling relationships with whole organismal metabolic rates.

## Materials and Methods

### Fish care

Laboratory-reared *D*. *rerio* (5D zebrafish; founder fish provided by Dr. Heather Stapleton, Duke University), *O*. *latipes* (Orange-red Japanese medaka; kindly provided by Dr. David Hinton, Duke University) were maintained in a recirculating AHAB system (Aquatic Habitats, Inc., Apopka, FL, USA). Juvenile *D*. *rerio* were fed Ziegler’s Larval AP100 (Zeigler Bros., Inc., Gardners, PA, USA) and adults received a mix of Cyclop-eeze (Argent Chemical Laboratories, Inc., WA, USA) and Zeigler’s Adult Zebrafish Complete Diet. *O*. *latipes* were fed Otohime B1 diet (Aquatic Eco-Systems, Inc., Apopka, FL, USA). *P*. *promelas* (Aquatic Biosystems, Fort Collins, CO, USA) were maintained in aerated 50 L aquaria and were fed Aquatox Flake Fish Food (Aquatic Eco-Systems, Inc., Apopka, FL, USA). All animal handling was approved by the Duke University Institutional Animal Care & Use Committee.

*F*. *heteroclitus* and *G*. *holbrooki* were collected with dip nets from King’s Creek (37°18′16.2″N, 76° 24′58.9″W), a tributary of the Severn River, VA (Permit No:15–003; 60–70 fish were collected per species). After transport to the laboratory, fish were maintained in a recirculating AHAB system (Aquatic Habitats, Inc., Apopka, FL, USA) in 15 ppt artificial seawater (ASW; Instant Ocean, Foster & Smith, Rhinelander, WI, USA). Fish received a mix of Cyclop-eeze (Argent Chemical Laboratories, Inc., WA, USA) and Zeigler’s Adult Zebrafish Complete Diet (Zeigler Bros., Inc., Gardners, PA, USA). Fish were acclimated to laboratory conditions for 2–3 months. All five species were fed *ad libitum* and maintained between 28–29°C with a 14:10 h light-dark cycle.

Frequency distributions of the sizes of fish from each species used for the respirometry analysis and subsequent tissue OCR measurements are in S1 Table and S1 Fig. Only male fish were used in the study; however, gender identification was difficult in small juveniles. It should be noted that for a given species, the size range of specimens used represented different life history stages.

### Respirometry measurements

RMO_2_ measurements were obtained across a range of sizes for each teleost species using two mini 170 mL swim tunnel respirometers (Loligo Systems, Tjele, Denmark) equipped with fiber optic oxygen transmitters (PreSens–Precision Sensing GmbH, Regensburg, Germany). The respirometers were bleached daily to minimize bacterial growth, and background OCR measurements revealed negligible values.

Each fish was isolated 24 h prior to the experiment and were not allowed to feed. Experiments were conducted at 28°C. The fish (*D*. *rerio* (n = 48), *F*. *heteroclitus* (n = 28), *G*. *holbrooki* (n = 25), *O*. *latipes* (n = 30), and *P*. *promelas* (n = 12)) were allowed to acclimate in the swim tunnel for 1 h prior to the first measurement—preliminary experiments using 5–6 *F*. *heteroclitus* and *D*. *rerio* suggested that 1 h acclimation time was sufficient to obtain RMO_2_. Following the acclimation period, four oxygen consumption measurements were taken at 15 min intervals for another 1 h. For each measurement, the tunnel was sealed and oxygen concentrations (mg L^-1^) over 5 min were recorded. OCR (mgO_2_ h^-1^) was calculated for each 5 min measurement window, and the lowest measured OCR was determined to be RMO_2_.

MMO_2_ measurements were also obtained for *D*. *rerio* by increasing the swim tunnel motor speed until the fish could no longer swim against the current. The speed was then slowly decreased to find the maximum speed at which the fish could maintain swimming, and the fish were swum until exhaustion (defined as when the fish stopped swimming against the current, fell against the back baffle, and did not attempt to swim away). Change in oxygen concentration (mg L^-1^) was recorded as above to determine MMO_2_ (mgO_2_ h^-1^).

Following respirometry measurements, fish were placed back on the recirculating AHAB system in individual 1 L tanks to allow for comparison of their tissue OCR with their corresponding RMO_2_ for all the species and MMO_2_ for *D*. *rerio*. The fish were allowed to recover for 7–8 days prior to sacrifice for tissue OCR measurements.

### Tissue specific OCR using XF^e^24 Extracellular Flux Analyzer

Each individual fish was anesthetized in ice water, weighed, and euthanized by cervical dislocation. The full heart was removed by the bulbus arteriosus, and the skull was carefully shaved away to remove the whole brain by the brainstem. The full liver was carefully removed without any additional connective tissue. Dissection of tissues was completed in less than two minutes per fish. Immediately after isolation, each tissue was weighed, washed in a Ringer’s solution (8.4 mmol l^-1^ sodium pyruvate, 5.6 mmol l^−1^ NaHCO_3_, 0.97 mmol l^−1^ HEPES, and 3.2 mmol l^−1^ HEPES sodium salt was added fresh to a stock of 115 mmol l^−1^ NaCl, 2.7 mmol l^−1^ KCl, 1.2 mmol l^−1^ MgCl_2_, 0.64 mmol l^−1^ NaH_2_PO4, and 2.1 mmol l^−1^ CaCl_2,_ pH 7.0 at 28°C) adapted from Little and Seebacher [10]), and placed in 525 μL Ringer’s in separate wells of an XF24 islet capture plate (Seahorse Bioscience, Billerica, MA, USA). During method development, we tested several Ringer’s solution recipes including Krebs-Ringer’s solution described by Kitambi and colleagues [17] who maintained heart tissues *ex vivo* for 4 days. However, we found the Ringer’s solution adapted from Little and Seebacher [10] yielded the most consistent OCR data.

Once plated, the tissues were confined to the bottoms of the wells using islet plate capture screens. Two to four wells per plate did not contain tissues and served as temperature controls. After completing the dissections, Ringer’s solution in each well was refreshed, maintaining the volume at 525 μL. The plate was incubated at 28°C for 30–45 min prior to the analysis by the XF^e^24 Extracellular Flux Analyzer instrument. For pharmacological inhibition, 75 μL of the desired agents (as described below) were loaded into the injection ports as per manufacturer’s instructions. At least eight measurement cycles consisting of 1 min mix, 1 min wait, and 2 min measure periods were taken to establish basal rates. Using this method, stable and consistent basal measurements were obtained for up to 4 h in preliminary experiments. A 1 min mix and 1 min wait cycle was used to re-oxygenate the wells prior to each pharmacological agent injection. Preliminary experiments were conducted to determine the mass of each tissue needed to achieve OCRs within the dynamic range of the XF^e^24 instrument.

### Metabolic partitioning with pharmacological agents

Pharmacological agents oligomycin, carbonyl cyanide 4-(trifluoromethoxy) phenylhydrazone (FCCP), sodium azide, N,N'- dicyclohexylmethanediimine (DCCD), antimycin A, and rotenone were obtained from Sigma-Aldrich (St. Louis, MO, USA). Experimental trials were conducted for each drug per fish species, size class, and tissue type to identify the drug concentrations that produced the maximum change in respiration (i.e. the “working concentration” (Table 1)), the optimum number of cycles, and the mix/wait/measure times that produced the least variable measurements (S2 Table). Preliminary experiments also showed that OCR rates for FCCP treatment following oligomycin treatment were lower and more variable than those obtained for FCCP treatment alone. However, OCR rates for sodium azide or the mix of antimycin A + rotenone treatment did not depend on the pretreatments. Thus, FCCP and oligomycin measurements were done in separate analyses, each coupled with sodium azide or antimycin A + rotenone treatment to allow for comparison.

**Table 1:** Working concentrations and mechanisms of action for each of the pharmacological agents that yielded the highest percent change in OCR compared to basal OCR for *D*. *rerio* and *F*. *heteroclitus*.

Compound	Mechanism of Action	Working concentration (mM) for *Danio rerio*	Working concentration (mM) for *Fundulus heteroclitus*
**FCCP**	Protonophore and un-coupler of oxidative phosphorylation, depolarizes the mitochondrial membrane	0.002 in1% DMSO	0.005
**Sodium azide**	Inhibits cytochrome c oxidase (Complex IV)	100^a^	30^a^
**Antimycin A + rotenone**	Antimycin A: inhibits cytochrome c reductase (Complex III), Rotenone: inhibits NADH dehydrogenase (Complex I) (This mixture can be used instead of sodium azide)	0.044 + 0.044	0.044 + 0.044
**Oligomycin**	Inhibits the proton channel of ATP synthase (Complex V), blocking oxidative phosphorylation of ADP to ATP	0.010 in 1% DMSO	0.015–0.025 in 1% DMSO^a^

^a^At these concentrations oligomycin and NaN_3_ yielded a ~30% decrease in OCR compared to basal OCR.

To compare *D*. *rerio* and *F*. *heteroclitus* cardiac bioenergetic profiles, the three lowest basal OCR measurements from each species were averaged to estimate total basal respiration (including mitochondrial and non-mitochondrial respiration). To obtain change in OCR due to pharmacological agents, the three highest OCR measurements were averaged post FCCP treatment, and the three lowest OCR measurements were averaged for sodium azide or antimycin A + rotenone treatment. Not all the specimens were subjected to every drug treatment, and average values across a group were used for final calculations. As the pharmacological inhibitors (sodium azide excepted) were prepared in DMSO, we measured tissue OCR following injection of only DMSO to confirm that treatment with up to 2% DMSO did not change OCR.

### Tissue Specific ECAR using the XF^e^24 Extracellular Flux Analyzer

To obtain basal extracellular acidification rate measurements (ECAR), a modified un-buffered assay solution was prepared by only adding the sodium pyruvate to the Ringer’s stock as described above (buffered solutions interfere with the pH measurements by the pH sensor in the cartridge). An aliquot of this solution was used to prepare a buffering solution containing 8X concentrated NaHCO_3_, HEPES, and HEPES sodium salt. Both solutions were also adjusted to pH 7.0 at 28°C.

For ECAR analysis, the tissues were plated in 525 μL of the modified un-buffered Ringer’s solution and 75 μL of the 8X concentrated buffering solution were loaded into the injection ports. Ten measurement cycles consisting of 1 min mix, 1 min wait, and 2 min measure periods were taken to establish basal ECAR. Subsequently, the buffering solution was injected into the wells to restore the original Ringer’s solution composition. Another 12 measurement cycles were taken to establish basal OCR. The ECAR measurements that were obtained using the un-buffered Ringer’s solution pre- and post-injection of buffering components, were then plotted against corresponding OCR measurements for *D*. *rerio* heart and brain tissues.

### Data and statistical analyses

Whole fish and tissue specific OCR values (mgO_2_ h^-1^) were log_10_ transformed and plotted against log of body mass to calculate the slope *b* from the equation *Y = a M* ^*b*^ (*log Y = log a + b log M*). Initially a nonlinear regression model was conducted to test for linearity of the data. Subsequently, a simple linear regression that allows for outlier elimination and comparison of slope (*b*) was performed. Segmental nonlinear regression analysis was performed for log brain OCR against log brain mass and log body mass to determine any size-specific scaling effects. Slopes and intercepts of linear regressions were compared based on exact sum of squares F test (equivalent to analysis of covariance [18]) for all five species as a group and per individual species. To account for repeated measures and further test the statistical significance of the similarity of heart OCR and organismal RMO_2_ scaling exponents, log ratio of heart OCR to organismal RMO_2_ was plotted against log of body mass, and the slope of this relationship is compared to zero. *P* values for each individual species obtained from this analysis was used to calculate a global *P* value using Fisher's combined probability test to confirm the similarity in scaling relationship (if *P>0*.*05*) between heart OCR and whole organismal OCR with body mass across all species tested. To compare the relationship between heart OCR and whole fish RMO_2_, a Spearman correlation coefficient was calculated with a two-tailed probability test for all five species combined and for each individual species. To assess *D*. *rerio* maximum metabolic rate scaling relationship between heart and whole organisms and with basal rates, taking repeated measures into account, two additional pairwise t-tests were conducted comparing (1) ratios of basal heart OCR: organismal RMO_2_ with maximal heart OCR: organismal MMO_2_ and (2) ratios of heart basal OCR: maximal OCR with organismal RMO_2_: MMO_2_.

Heart specific OCR data obtained for metabolic partitioning using pharmacological agents were corrected for size (per 0.001 g of heart) using the scaling exponent derived for each species and are presented as mgO_2_ h^-1^. Statistical significance was tested using Two-Way ANOVA followed by Tukey’s post-hoc test to correct for multiple comparisons (significance was accepted if *p<0*.*05*). All the analyses were conducted using Graph Pad Prism 6.0 (San Diego, CA, USA).

## Results

### Organismal respiration and basal tissue-specific OCR

**Fig 1:**
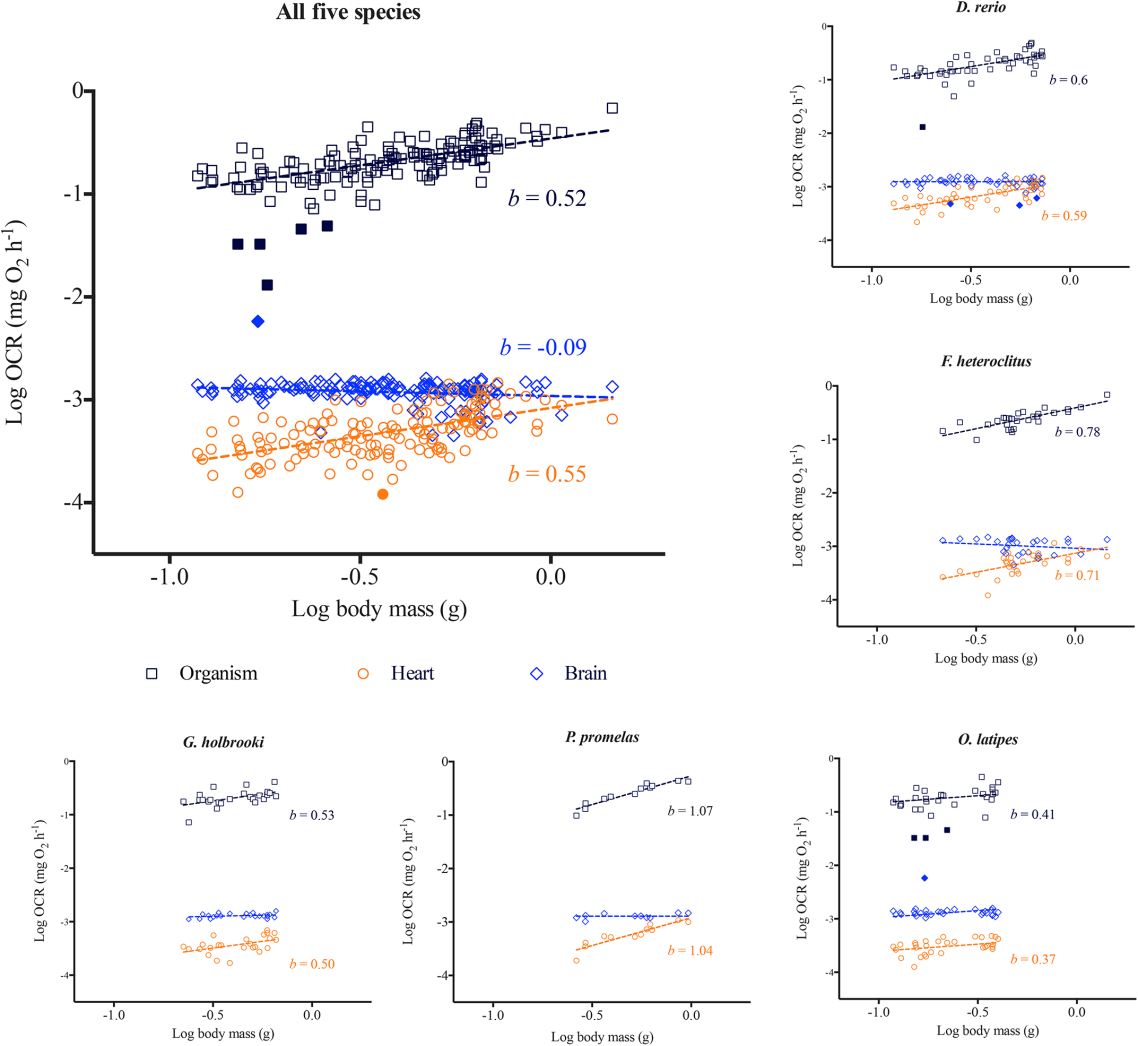
Whole organismal, heart and brain oxygen consumption rate (OCR) scaling relationship with body mass. Log of oxygen consumption rates measured for whole organisms and for isolated hearts and brains from each fish are plotted against log of body mass for (a) all five species *(n = 143)*, (b) *Danio rerio (n = 48)*, (c) *Fundulus heteroclitus (n = 28)*, (d) *Gambusia holbrooki (n = 25)*, (e) *Oryzias latipes (n = 30)*, and (f) *Pimephales promelas (n = 12)*. Regressions were conducted across each data set based on least-squares test. *b* is the scaling exponent. Standard error, 95% confidence interval and *R* ^*2*^ values and the scaling exponent *b* for brains for individual species are included in Table 2 and S3 and S4 Tables. Filled symbols represent outliers determined by the linear regression model.

**Fig 2:**
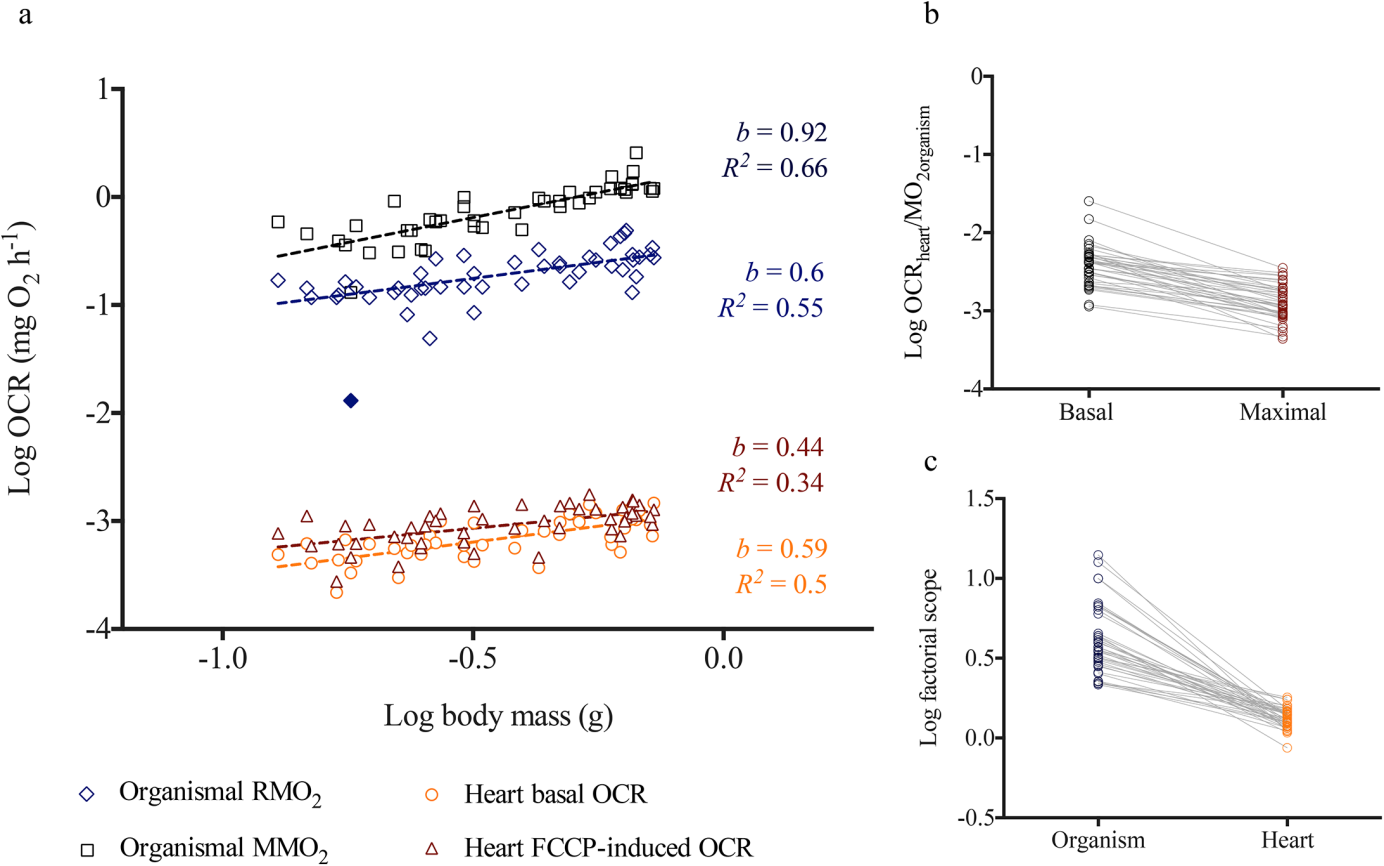
Metabolic scaling relationship between basal and maximal oxygen consumption rates (OCR) for *Danio rerio*. (a) Log transformed values of maximal (MMO_2_) and routine (RMO_2_) oxygen consumption rates (OCR) measured for whole organisms and basal and FCCP induced OCR for isolated hearts are plotted against log of body mass (*n = 45*). Regressions were conducted across each data set based on least-squares test. Filled symbols represent outliers determined by the linear regression model. *b* is the scaling exponent. (b) Paired t-test comparing ratios of log basal heart OCR: organismal RMO_2_ with log maximal heart OCR: organismal MMO_2_. (c) Paired t-test comparing ratios of log heart basal OCR: FCCP-induced maximal OCR with log organismal RMO_2_: organismal MMO_2_.

Whole organismal RMO_2_ for all five species demonstrated a classic allometric scaling relationship with body mass (Fig 1). The RMO_2_ scaling exponent *b* for the five species combined was 0.52. However, this value was species-specific where, *b* = 0.60 for *D*. *rerio*, *b* = 0.53 for *G*. *holbrooki*, *b* = 0.41 for *O*. *latipes*, and was higher for *F*. *heteroclitus* (0.78) and *P*. *promelas* (1.07) (Fig 1; Table 2) (extended statistical information included in S3 Table). In *D*. *rerio*, maximum metabolic rate (MMO_2_) *(b =* 0.92) scaled differently from RMO_2_ *(b =* 0.60) (Fig 2A).

**Table 2:** Slopes (exponent *b*) and *R* ^*2*^ values for log oxygen consumption rates of whole organisms, hearts and brains *vs* log body mass for all five species combined and for each individual species^1^.

		Organism	Heart	Brain
**All five species**	Slope ± Std. Err.	**0.52** ± 0.05	**0.55** ± 0.06^a^	-0.09 ± 0.04
	*R* ^*2*^	0.44	0.34	0.04
***Danio rerio***	Slope ± Std. Err.	**0.60** ± 0.1006	**0.59** ± 0.09^a^	-0.03 ± 0.04
	*R* ^*2*^	0.44	0.51	0.01
***Fundulus heteroclitus***	Slope ± Std. Err.	**0.78** ± 0.12	**0.71** ± 0.18^a^	-0.16 ± 0.16
	*R* ^*2*^	0.62	0.38	0.04
***Gambusia holbrooki***	Slope ± Std. Err.	**0.53** ± 0.17	**0.50** ± 0.20^a^	0.08 ± 0.06^a^
	*R* ^*2*^	0.29	0.23	0.08
***Oryzias latipes***	Slope ± Std. Err.	**0.41** ± 0.17	**0.37** ± 0.13^a^	0.02 ± 0.05
	*R* ^*2*^	0.18	0.23	0.01
***Pimephales promelas***	Slope ± Std. Err.	**1.07** ± 0.11	**1.04** ± 0.15^a^	0.13 ± 0.08
	*R* ^*2*^	0.90	0.83	0.28

^1^See S3 Table for Y intercept values, 95% confidence intervals and sample sizes.

^a^Exponent *b* for hearts or brains are statistically similar (*P>0*.*05*) to that of whole organisms (*F (DFn*, *DFd*) and *P* values are in S4 Table).

**Fig 3:**
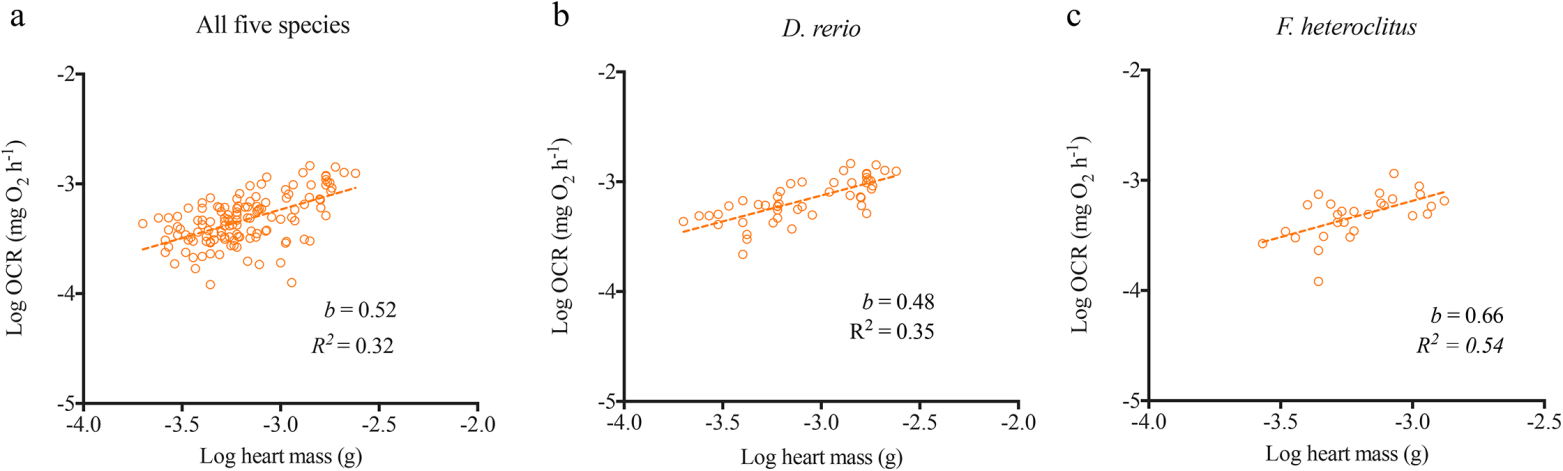
Heart metabolic scaling relationship with heart size. Log of basal oxygen consumption rates (OCR) for isolated hearts is plotted against log of isolated heart mass for (a) all five species *(n = 130)*, (b) *Danio rerio (n = 48)* and (c) *Fundulus heteroclitus (n = 28)*. Non-linear regressions were conducted across each data set based on least-squares test. *b* is the scaling exponent. Standard error, 95% confidence interval and *R* ^*2*^ values and the scaling exponent *b* for individual species are included in S5 Table.

Using the XF^e^24 Extracellular Flux Analyzer, we successfully obtained basal OCR for heart and brain tissues in all five species and liver tissues in *D*. *rerio* and *F*. *heteroclitus* (S2 Fig). Total basal OCR for hearts increased allometrically with increasing heart mass (*b =* 0.52) as well as mass of whole organism (*b =* 0.55) for the five species combined and for each individual species (Figs 2 and 3; Tables 2 and 3; See S3–S5 Tables for detailed statistical information). This metabolic scaling exponent with body mass was species-specific and was higher for *F*. *heteroclitus* (*b =* 0.71) and *P*. *promelas* (*b =* 1.04) compared to the other three species; *b* was 0.59, 0.50 and 0.37 for *D*. *rerio*, *G*. *holbrooki*, and *O*. *latipes*, respectively (Fig 1; Table 2).

**Table 3:** Slopes and Y intercepts for log_10_ oxygen consumption rates for hearts plotted against log_10_ heart mass for all five species and for each *Danio rerio and Fundulus heteroclitus*.

	All five species	*Danio rerio*	*Fundulus heteroclitus*
**Slope (*b)***	0.5196	0.4722	0.6588
**Y Intercept**	-1.675	-1.709	-1.210
**Std. Error**			
**Slope**	0.06771	0.06381	0.1781
**Y Intercept**	0.2161	0.1976	0.5718
**95% Confidence Intervals**			
**Slope**	0.3869 to 0.6523	0.3437 to 0.6008	0.2926 to 1.025
**Y Intercept**	-2.099 to -1.252	-2.107 to -1.311	-2.385 to -0.034
**Goodness of Fit**			
**R** ^**2**^	0.3151	0.5435	0.3448
**Sample size** ^**a**^			
**Analyzed**	130	48	28
**Outliers**	0	0	0

^a^Total number of fish tested is obtained by combining number of samples in analyzed and outliers rows.

Comparisons of the slopes of the logarithmic relationships between whole organismal OCR with body mass and heart OCR with body mass showed equivalent values that were deemed statistically similar for all five species combined and for each individual species (Fig 1; Table 2; S3 and S4 Tables). This relationship was consistent across all the species, indicating that heart tissue metabolic rates and whole organismal metabolic rates scale similarly with body mass in a species-specific fashion (χ^2^ *= 13*.*18*, *P = 0*.*2136 –*based on Fisher's combined probability test; see S4 Table for species specific statistical information). Spearman correlation coefficient for log transformed whole animal OCR *vs* heart OCR for all five species combined was 0.40 (*P<0*.*0001*), and 0.48 for *D*. *rerio*, 0.40 for *F*. *heteroclitus*, 0.2 for *G*. *holbrooki*, 0.48 for *O*. *latipes*, and 0.96 for *P*. *promelas*. In contrast to basal or routine rates, organismal MMO_2_ (*b = 0*.*92*) and FCCP-induced maximal heart OCR *(b = 0*.*44)* in *D*. *rerio* scaled differently (Fig 2A). Paired t-tests indicated that the relationship between basal heart OCR and RMO_2_ is significantly different (*P>0*.*0001*) from that of maximal heart OCR and MMO_2_ (Fig 2B). Cardiac factorial scope (ratio between maximal and basal OCR) also significantly differed (*P>0*.*0001*) from organismal factorial scope (Fig 2C) across different body sizes. These findings suggest that metabolic scaling relationships between heart and whole organisms are only maintained under routine or basal conditions, potentially due to altered physiological responses with increasing metabolic demand.

**Fig 4:**
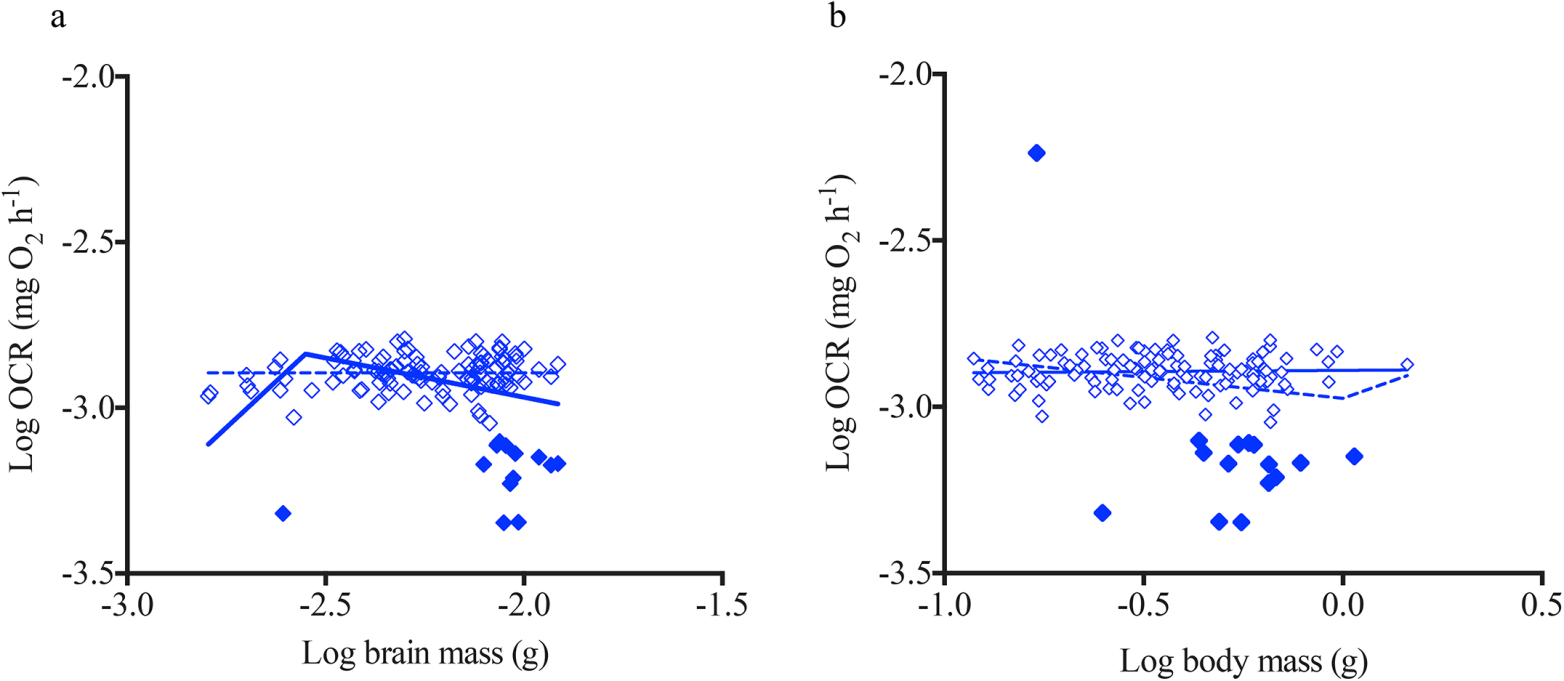
Brain metabolic scaling relationship with size. Log of basal oxygen consumption rate (OCR) for isolated brains is plotted against (a) log of isolated brain mass and (b) log of body size for all five species *(n = 130)*. Non-linear straight line and segmental regressions were conducted across each data set based on least-squares test. Filled symbols represent outliers determined by the linear regression model.

In contrast to hearts, brain OCR did not increase with increasing brain mass or body mass, as demonstrated by a scaling exponent ~0 (Figs 1 and 4; Table 2). A two segmented linear regression analysis showed that metabolic rates of smaller brains (*n = 10*) increased with increasing brain mass; however, such a relationship was not detected when plotted against body mass (Fig 4). Heart and brain mass increased with increasing body size, but the proportional increase in organ mass with body mass was smaller for brains compared to hearts with body mass (S3 Fig).

### Metabolic partitioning of OCR in tissues

**Fig 5:**
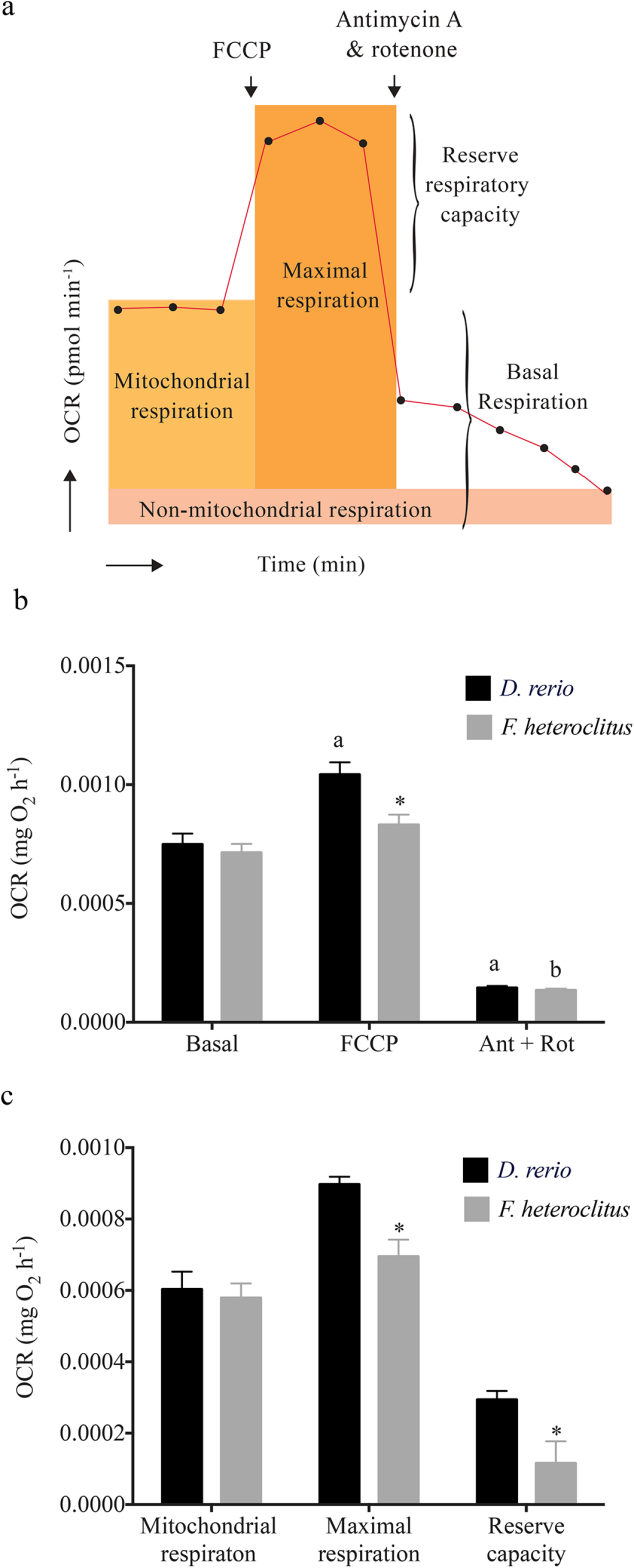
Metabolic partitioning of *Danio rerio* and *Fundulus heteroclitus* heart tissue oxygen consumption rate (OCR). (a) Conceptual diagram depicting use of FCCP and antimycin A + rotenone and the metabolic parameters calculated from changes in oxygen consumption. (b) OCR following exposure to FCCP and antimycin A + rotenone (Ant + Rot). (c) Total OCR due to mitochondrial respiration, total mitochondrial capacity and mitochondrial reserve capacity calculated based on FCCP and antimycin A + rotenone. ‘a’ denotes statistical significance compared to *D*. *rerio* basal heart OCR and ‘b’ denotes statistical significance compared to *F*. *heteroclitus* basal heart OCR. Statistical significance between *D*. *rerio* and *F*. *heteroclitus* for a given measurement is denoted by * (Two-Way ANOVA followed by Tukey’s post-hoc test to correct for multiple comparisons; *P<0*.*05*). Values are expressed as means ± S.E.M.

Based on a series of titration experiments, we were able to develop “working concentrations” for carbonyl cyanide 4-(trifluoromethoxy) phenylhydrazone (FCCP) antimycin A + rotenone and successfully utilize these compounds to partition total basal OCR into its component fractions for heart tissues in *F*. *heteroclitus* and *D*. *rerio* (Fig 5; Table 1). Oligomycin decreased OCR, providing an index for the fraction of total basal respiration that is due to ATP production. However the percent decrease was only ~30% of basal OCR for both the species (data not shown), which is unlikely to be representative of total OCR due to ATP production *in vivo*. However, under assaying conditions where ATP demand and turnover is low, ATP synthase activity might be lower, resulting in only a ~30% decrease in OCR [19]. Titration data also showed that compared to sodium azide, antimycin A + rotenone is a better inhibitor of mitochondrial function in tissues, leading to a decrease of ~80%, approximating the fraction of total basal respiration that is non-mitochondrial in *F*. *heteroclitus and D*. *rerio* hearts. The uncoupling agent FCCP led to an increase in OCR of 16% and 40% for *F*. *heteroclitus* and *D*. *rerio*, respectively. The difference between the maximal respiration following FCCP treatment and the total basal respiration provides an index of the mitochondrial reserve capacity of heart tissues. Maximal FCCP-uncoupled OCR was determined by calculating the difference between OCR after inhibition by antimycin A + rotenone and OCR due to FCCP response (Fig 5). Comparison of metabolic partitioning data between *F*. *heteroclitus* and *D*. *rerio* show that while basal OCR per 0.001g of heart for both species are similar, *D*. *rerio* hearts have higher reserve capacity and maximal mitochondrial capacity compared to *F*. *heteroclitus* (Fig 5). Our attempts to partition components of brain and liver OCR in *F*. *heteroclitus* and *D*. *rerio* were less successful.

### Metabolic profiles of heart and brain tissues

**Fig 6:**
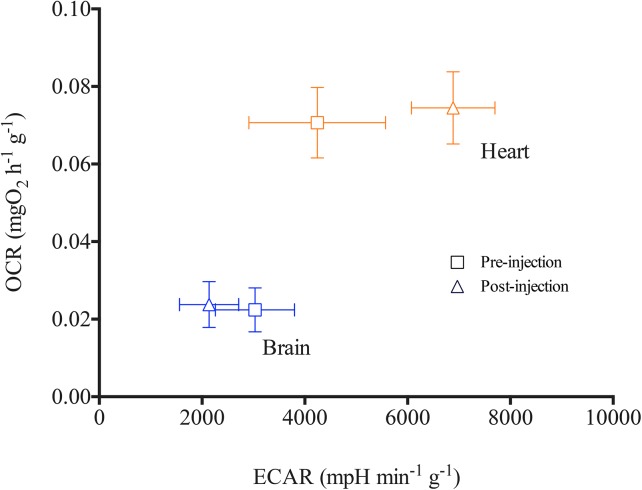
Metabolic profiles of *Danio rerio* heart and brain tissues. Oxygen consumption rate (OCR) calculated per gram of tissues is plotted against extracellular acidification rates (ECAR) *(n = 5)*. Measurements recorded in un-buffered solution are shown in square symbols and triangles represent values post-injection of buffering components of the Ringer’s solution. Values are expressed as means ± S.E.M.

Metabolic profiles indicative of aerobic and anaerobic status of *D*. *rerio* heart and brain tissues are demonstrated in Fig 6. Data show that ECAR values are less variable in the un-buffered solution compared to post-buffer injection. OCR measurements remain unchanged pre- and post-injection of buffered solution, confirming that oxygen consumption is not impacted by un-buffered Ringer’s for a short period of time (~25 min). While this approach provides an index for glycolysis based on ECAR, it should be noted that processes other than glycolysis can affect extra cellular H^+^ concentration and further studies are necessary to precisely characterize glycolytic rates of these tissues.

## Discussion

To our knowledge, this is the first examination of intact tissue-specific oxygen consumption rates in the context of allometric scaling relationship with body mass. For each species, scaling exponents of whole heart OCR were indistinguishable from the corresponding scaling exponent of whole animal metabolic rates with body mass. A similar relationship was not detected for brains. Here we discuss these data in detail and consider the potential implications and caveats of the high-throughput tissues specific *ex vivo* mitochondrial function analysis assay that we utilized in the current study.

### Allometric metabolic scaling of hearts and whole organisms

The biological significance of allometry, particularly the scaling relationship of organismal metabolic rate with body size, has been widely studied for almost two centuries. Based on a large data set consisting of a number of species across varying sizes, Kleiber [20] determined an approximate 0.75 exponent (*b*) to describe the relationship between body mass and basal metabolic rate. In a series of studies, West and colleagues [6–9] provided strong evidence to support this ¼ power scaling relationship for metabolic rates and described how the underlying factor driving this relationship is the fractal nature of the distributing vascular network of an organism. However, the scaling exponent *b* remains contentious and highly debated in the literature, with studies presenting evidence to support a geometric scaling exponent of 0.66—a value derived from surface area to volume ratio of an organism [21–23]. In the current study, we only found *F*. *heteroclitus* RMO_2_ to demonstrate scaling exponent similar to 0.75, while other species showed values that ranged from 0.4–1. For these species, it is difficult to speculate the potential factors underlying the scaling exponents that are different from 0.75. Considering that *G*. *holbrooki* and *F*. *heteroclitus* demonstrate different scaling exponents but are captured from exactly the same estuarine habitat, it is implausible that the differences in their scaling relationships are driven by environmental conditions. The scaling exponent of 0.6 for *D*. *rerio* was particularly unexpected considering that Lucas and colleagues [24] showed a value of 0.96 for *b* in a study that examined juvenile and adult *D*. *rerio* across a similar range of sizes at the same temperature using comparable methods, except for the tank acclimation period prior to measurement. Lucas and colleagues [24] measured standard metabolic rate in fish that were left in the respirometer for 48 h, whereas in the current study we only acclimated fish for 2 h. This coupled with *D*. *rerio* strain specific differences and varying rearing conditions may explain the lower scaling exponent detected in the current study for this species. More importantly, the scaling exponent of 0.75 is often associated with basal metabolic rates, and in the current study, the organismal metabolic rates we measured are most likely to represent routine metabolic rates. Nonetheless, despite numerous studies supporting 0.75 as the universal scaling exponent, other studies have found a range of values (0.4–1) for this exponent in fish and is attribute this to differences in lifestyle, swimming mode and environmental characteristics of the habitat [25,26], as well as the conditions in which the data are collected [22].

Despite detecting scaling exponents that contradict the 0.75 scaling law for some of the fishes in the current study, the scaling exponents of the heart-specific OCR and corresponding routine organismal metabolic rate were nearly identical for each species. According to West and colleagues [6,9], proportionate resource distribution within an organism by a fractal-like vascular network is the fundamental tenant underlying the metabolic scaling exponent with body size. It is conceivable that the heart, as the power generator of this vascular distribution network, plays an important role in determining the organismal metabolic scaling relationship with body mass. This hypothesis is further supported by the findings that total mitochondrial volume and citrate synthase activity—thus, aerobic metabolism of heart tissues—are predictive of and proportional to resting cardiac power output across a range of vertebrate animals [27,28] including in fish. Therefore, considering the indistinguishable metabolic scaling exponent demonstrated by hearts and corresponding teleost species with body mass, it is possible that metabolic rate of the heart defines the scaling exponent for whole organismal metabolic rate under routine conditions, at least in ectothermic teleosts. However, we caution that similarity in the scaling relationship in heart and whole organismal metabolic rates may not imply a physiological functional connection between the two.

In contrast to basal or routine rates, organismal MMO_2_ (*b = 0*.*92*) and FCCP-induced maximal heart OCR *(b = 0*.*44)* in *D*. *rerio* scaled differently. As discussed by Weibel and Hoppeler [29], maximum metabolic rate might be determined by the ATP demand of cells that are active during work in mammals. Thus, during maximal swimming activity, skeletal muscle, rather than cardiac muscle, demand for ATP might dictate OCR in *D*. *rerio* and may explain the different scaling exponents detected for organismal MMO_2_ and heart maximal OCR. However, compared to mammals, teleost skeletal muscle is less oxidative [30] and is difficult to speculate how this may affect MMO_2_ scaling exponent in fish. In addition, it is possible that FCCP-induced maximal heart OCR is underestimated in the current study as a result of substrate limitation. Glucose as well as fatty acids are primary fuel sources for cardiac muscle [31]. Further studies are necessary to investigate the effect of different metabolic fuels sources on maximal OCR of hearts.

### Brain metabolism and body size

In contrast to hearts, the brain OCR data suggest that not all tissues follow the same scaling relationship within an organism. However, Somero and Childers [32], showed that citrate synthase enzyme activities of teleost brain tissues demonstrate an allometric scaling relationship with body size, predicting a similar relationship for OCR for whole brains with body size. This finding is consistent with observed scaling relationships for mammalian brains, where the scaling exponent of OCR for different components of the brain, except for the brain stem, was ~0.86 [33,34]. Since brains are highly aerobic tissues, it has also been proposed that oxygen demand by brain tissues might be a significant driver of whole organismal OCR. However, this hypothesis remains inconclusive [35]. Nonetheless, these studies suggest a strong relationship between body size, whole animal metabolic rates and brain OCR, which was not detected in the current study.

Several factors may underlie the absence of an allometric scaling relationship we detected for brains. The scaling exponent for the brain stem is ~0, a value similar to that observed for whole brains in the current study, whereas that of the rest of the brain is 0.86 in mammals [33]. During tissue excision, fish brains were removed by the brainstem, inclusion of which may have driven the scaling exponent of the whole brain toward zero. In addition, the brain samples used in the current study are ~5–20x larger (in mass and volume) than hearts (S3 Fig). Therefore, it is possible that difference in surface area to volume ratios of brains compared to hearts affected oxygen diffusion across brain tissues. This hypothesis is supported by the segmental linear regression demonstrating that smaller brains may scale allometrically with brain mass; however, this relationship was only defined by data for ten samples and was not detected with whole body mass (Fig 4). In addition, smaller fish were younger in age, and the cellular composition of the brain tissues might be different from that of older specimens. Thus, further research quantifying OCR in whole brains from smaller teleosts is necessary to obtain conclusive evidence on scaling relationship of teleost brain metabolic rates. In addition, this research should focus on how the metabolic activity of different brain components varies with the developmental stage or due to differences in cellular composition [36,37] and how these factors contribute to metabolic scaling in teleost brains.

### Potential implications of high throughput tissue bioenergetic assay

Despite not detecting a scaling relationship with body mass for brains, the current approach to measure heart, brain and liver bioenergetics has many potential applications. Examining rates and mechanisms of organismal energy metabolism extends into all aspects of biological research ranging from clinical studies to ecological analyses of organismal fitness. Mitochondria-derived injuries to cardiac, brain and liver tissues are widely studied, and mitochondrial dysfunction is associated with pathogenesis of cardiac diseases [38], neurodegenerative diseases (e.g. Parkinson’s and Alzheimer’s Disease) [39,40] and liver injury [41]. Mitochondrial dysfunction is also studied in the contexts of various cancers, diabetes, ageing research and other metabolic syndromes (see review by Meyer and colleagues [42]). The approach described here allows for comparisons of changes in tissue-specific mitochondrial function *ex vivo* in these disease contexts, particularly using *O*. *latipes* and *D*. *rerio—*prominent biomedical and toxicological models, the latter of which is the premier study organism in cardiac regenerative biology [43] and considered a model system for mitochondrial biology and diseases [44].

In environmental biology and toxicology, mitochondrial function is assessed to identify effects of physical and environmental stressors on organismal fitness and survival. Chemical contaminants and physical environmental stressors can cause mitochondrial DNA and protein damage and structural perturbations [42,45]. Further, exposure to environmental stressors increases overall ATP demand, in order to induce an adequate stress response and restore cellular homeostasis, likely affecting mitochondrial function. Considering the rapid anthropogenic influences altering physical and chemical environmental factors, investigating their effects on mitochondrial integrity and understanding the role of mitochondria in organismal stress response are important areas of research in environmental biology and toxicology. The method we present here as well as the strong correlation between whole animal *vs* heart routine OCR that we discovered, provide a framework to evaluate overall organismal fitness and to compare shifts in metabolism following exposure to an environmental stressor, potentially by only measuring tissue-specific OCR.

### Use of pharmacological agents for metabolic partitioning

Despite the current approach providing a powerful platform to obtain tissue specific OCR with high sensitivity and reproducibility [10,11], the metabolic partitioning of tissue specific OCR was only successful in heart tissues of *D*. *rerio* and *F*. *heteroclitus*. As demonstrated in Fig 5, with the use of FCCP and antimycin + rotenone, we obtained indices for OCR due to (i) total mitochondrial and non-mitochondrial respiration, (ii) maximal mitochondrial respiration, and (iii) reserve respiratory capacity. Thus, further research is required to employ this method for brain and liver tissues and for the other model species. In addition, tissue-specific OCR upon pharmacological inhibition demonstrated a high degree of variability, even when corrected for organ mass based on the scaling relationship we developed here. This suggests that a high sample size is required to obtain statistically significant data. In fact we tested 28 or more samples per species to obtain species-specific statistically significant differences in change in OCR due to maximal respiratory capacity. It should be noted that each of the OCR values obtained using pharmacological agents are estimates, given that their mechanisms of action/inhibition are not 100% specific. This is particularly evident in the data for the decrease in OCR with oligomycin, as it is unlikely that only ~30% of oxygen consumed by heart tissues contributes to ATP synthesis and considering the high ATPase activity measured in fish heart tissues [46]. Nonetheless, judicious use of these pharmacological agents will likely enable a comparative analysis of changes in mitochondrial bioenergetics in fish hearts. Finally, the method we described to obtain tissue-specific extracellular acidification rates might provide further insights to anaerobic *vs* aerobic status in tissues of interest.

## Conclusion

Current data provide empirical evidence to show a strong relationship between cardiac oxygen consumption rates and organismal metabolic rates in the five teleost species tested. Furthermore, the current study is indicative of potential implications of the high-throughput approach to characterizing tissue-specific bioenergetics. As discussed by Kuznetsov and colleagues [16], *ex vivo* assessment of mitochondrial bioenergetics is more physiologically relevant than *in vitro* assessment. *Ex vivo* studies can yield consistent OCR data that accounts for experimental condition specific changes in cytosolic factors that affect mitochondrial function and potentially for heterogeneity in tissue specific mitochondrial copy number. In fact our attempts to measure OCR using the current method in hearts that were mechanically disrupted yielded highly variable results, suggesting the significance of intact tissue measurements. However, there are also obvious limitations to the current approach, such as the restriction of this method to teleost species of smaller size. In addition, the metabolic partitioning of tissue OCR is limited, and further research is necessary for extensive characterization of mitochondrial bioenergetics of intact tissues. Nonetheless, quantifying tissue-specific OCR has important implications in many contexts, including the discovery of identical scaling relationship between heart OCR and whole animal OCR with body mass for teleosts presented here.

## Supporting Information

S1 Fig Size distribution of specimens used. (EPS)

S2 Fig Basal oxygen consumption rate recording for *Danio rerio* heart, brain and liver tissues (Screen capture from XFe24 Extracellular Flux Analyzer), and change in OCR with injection of oligomycin followed by sodium azide. (PDF)

S3 Fig Log of heart and brain mass plotted against log of body mass for each individual species. (PDF)

S1 Table Specimen information. Mean and median of body mass, heart mass and brain mass used in the current study for each species.
(PDF)

S2 Table Pharmacological agent information. Different pharmacological agents used for partitioning metabolic function, the number of optimum measurement cycles, the concentrations tested, and the working concentrations for each agent, per tissue and per species.
(PDF)

S3 Table Detailed statistics on regression analyses. Slopes and Y intercepts for log oxygen consumption rates of whole organism, hearts and brains plotted against log body mass for all five species combined and for each individual species.
(PDF)

S4 Table Heart and brain scaling exponent comparison. Comparison of exponent b between log of heart and brain oxygen consumption rates (OCR) with log of whole organismal metabolic rates (RMO_2_). P and F values are calculated by testing if the slope of the curve log of tissue OCR:RMO_2_ plotted against log body mass is equal to zero.
(PDF)

S5 Table Detailed statistics for heart OCR versus heart mass. Slopes and Y intercepts for log oxygen consumption rates of hearts plotted against log heart mass for all five species combined and for *Danio rerio* and *Fundulus heteroclitus*.
(PDF)
