# Supplementary material for: High-Throughput Tissue Bioenergetics Analysis Reveals Identical Metabolic Allometric Scaling for Teleost Hearts and Whole Organisms
Source: PLoS One. 2015 Sep 14;10(9):e0137710. doi: 10.1371/journal.pone.0137710 (PMC4569437; doi:10.1371/journal.pone.0137710)

S2 Fig. Basal oxygen consumption rate recording for *Danio rerio* heart, brain and liver tissues (Screen capture from XFe24 Extracellular Flux Analyzer), and change in OCR with injection of oligomycin followed by sodium azide.

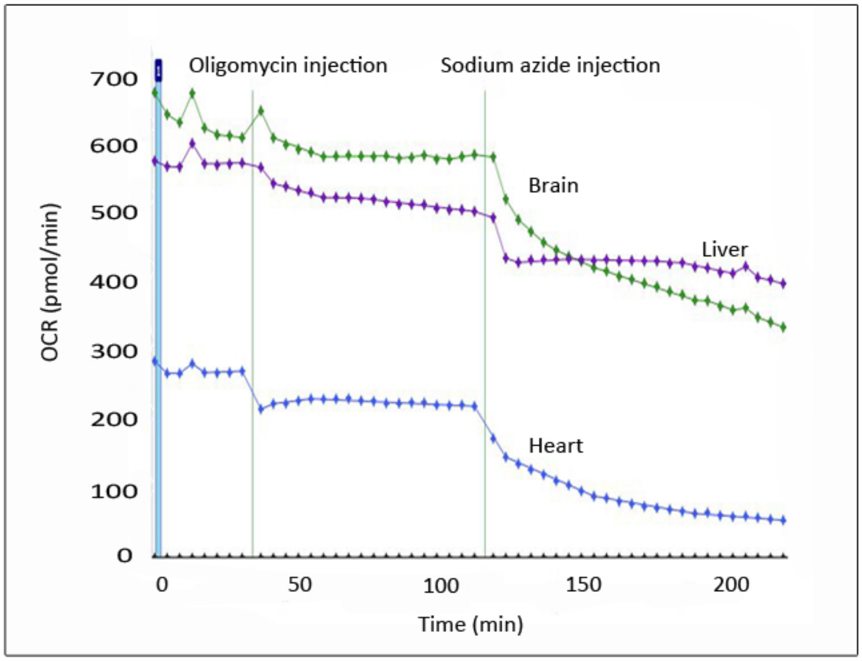

Supplement: S2 Fig — (PDF) [file pone.0137710.s002.pdf]
