# Supplementary figures and images for: High-Throughput Tissue Bioenergetics Analysis Reveals Identical Metabolic Allometric Scaling for Teleost Hearts and Whole Organisms
Source: PLoS One. 2015 Sep 14;10(9):e0137710. doi: 10.1371/journal.pone.0137710 (PMC4569437; doi:10.1371/journal.pone.0137710)

**S3 Fig.** Log of heart and brain mass plotted against log of body mass for each individual species.

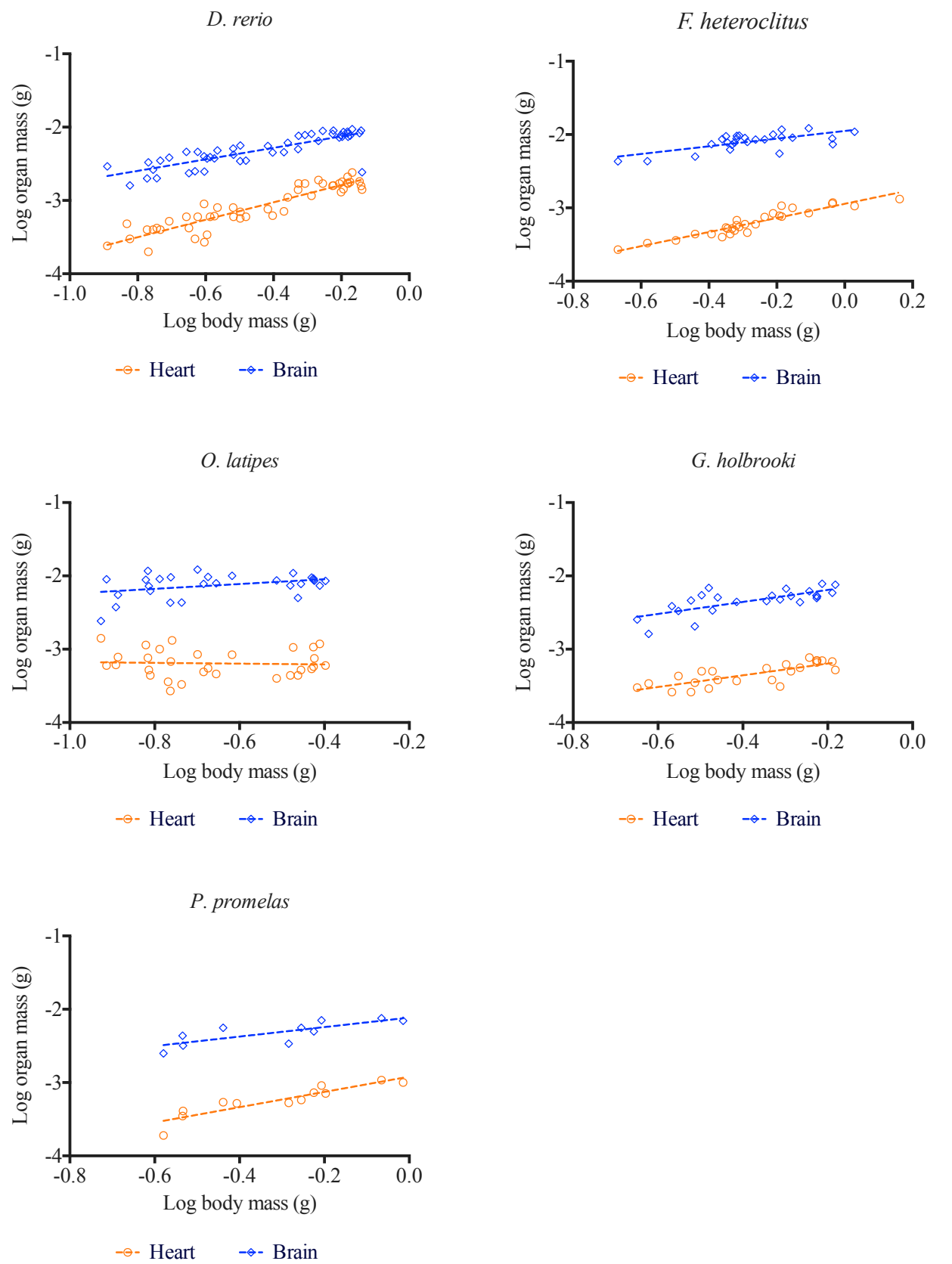

Supplement: S3 Fig — (PDF) [file pone.0137710.s003.pdf]
