# Supplementary material for: High-Throughput Tissue Bioenergetics Analysis Reveals Identical Metabolic Allometric Scaling for Teleost Hearts and Whole Organisms
Source: PLoS One. 2015 Sep 14;10(9):e0137710. doi: 10.1371/journal.pone.0137710 (PMC4569437; doi:10.1371/journal.pone.0137710)
Supplement: S1 Table — Mean and median of body mass, heart mass and brain mass used in the current study for each species. (PDF) [file pone.0137710.s004.pdf]

S1 Table. **Specimen information.** Mean and median of body mass, heart mass and brain mass used in the current study for each species.

		D. rerio	*O. latipes*	*P. promelas*	*F. heteroclitus*	*G. holbrooki*
**Fish (mg)**	Range	129.00-726.30 (n=48)	118.40-400.50 (n=30)	263.50-967.00 (n=12)	214.70-1450.70 (n=28)	224.30-657.70 (n=25)
	Mean	404.99	239.49	530.08	582.30	434.00
	Median	356.65	203.25	538.40	498.60	452.80
**Heart (mg)**	Range	0.20-2.40 (n=48)	0.17-0.84 (n=30)	0.19-1.08 (n=12)	0.27-1.32 (n=28)	0.26-0.77 (n=24)
	Mean	1.02	0.39	0.63	0.68	0.48
	Median	0.78	0.35	0.56	0.59	0.50
**Brain (mg)**	Range	1.60-9.40 (n=47)	1.77-5.36 (n=29)	2.50-7.54 (n=10)	4.30-12.18 (n=26)	1.62-7.81 (n=24)
	Mean	5.41	3.06	5.12	7.69	4.89
	Median	4.80	2.79	5.29	8.63	5.03
