# Supplementary material for: High-Throughput Tissue Bioenergetics Analysis Reveals Identical Metabolic Allometric Scaling for Teleost Hearts and Whole Organisms
Source: PLoS One. 2015 Sep 14;10(9):e0137710. doi: 10.1371/journal.pone.0137710 (PMC4569437; doi:10.1371/journal.pone.0137710)
Supplement: S2 Table — Different pharmacological agents used for partitioning metabolic function, the number of optimum measurement cycles, the concentrations tested, and the working concentrations for each agent, per tissue and per species. (PDF) [file pone.0137710.s005.pdf]

S2 Table. **Pharmacological agent information.** Different pharmacological agents used for partitioning metabolic function, the number of optimum measurement cycles, the concentrations tested, and the working concentrations for each agent, per tissue and per species.

Heart				
**Drug**	**Measurement cycles***	**Species**	**Range of concentrations tested**	**Working concentration**
Oligomycin	8-10	*D. rerio*	1-150 µM (up tp 1% DMSO)	10 µM in 1% DMSO
		F. heteroclitus	5-150 µM (up to 1% DMSO)	15-25 µM in 1% DMSO
		P. promelas	1-20 µM (up to 2% DMSO)	Not found
		O. latipes	1-20 µM	Not found
FCCP	6-8	*D. rerio*	0.5-150 µM (up to 1% DMSO)	2 µM in 1% DMSO
		F. heteroclitus	2-150 µM	5 µM
		P. promelas	2-20 µM (up to 2% DMSO)	Not found
		O. latipes	2-30 µM (up to 1% DMSO)	Not found
Sodium azide	25+	*D. rerio*	1-3000 mM (up to 1% DMSO)	100 mM
		F. heteroclitus	10 mM -100 mM	30 mM
		P. promelas	10-30 mM	Not found
		O. latipes	100 mM	Not found
Antimycin A + rotenone	25+	*D. rerio*	20-50 µM + 20-50 µM	44 µM + 44 µM
		F. heteroclitus	20-50 µM + 20-50 µM	44 µM + 44 µM
Rotenone	Not found	*D. rerio*	2-60 µM	Not found
DCCD	Not found	*D. rerio*	2-25 µM (up to 1% DMSO)	Not found
DMSO control	Not found	*D. rerio*	up to 2%	No effect
		P. promelas	up to 2%	No effect

Brain				
**Drug**	**Measurement cycles**	***Species***	**Range of concentrations tested**	**Final concentration**
Oligomycin	8-10	*D. rerio*	1-100 µM (up to 1% DMSO)	Not found
		F. heteroclitus	5-150 µM (up to 1% DMSO)	Not found
FCCP	6-8	*D. rerio*	2-150 µM (up to 1% DMSO)	Not found
		F. heteroclitus	2-150 µM	Not found
Sodium azide	25+	*D. rerio*	10-100 mM (up to 1% DMSO)	Not found
		F. heteroclitus	20-100 mM	Not found
Antimycin + rote	25+	*D. rerio*	50 µM M + 50 µM	Not found
		F. heteroclitus	25 mM + 25 mM	Not found
Rotenone	Not found	*D. rerio*	50-60 µM	Not found
DCCD	Not found	*D. rerio*	2-25 µM (1% DMSO)	Not found
DMSO control	Not found	*D. rerio*	up to 2%	No effect

		Liver		
**Drug**	**Measurement cycles**	**Species**	**Range of concentrations tested**	**Final concentration**
Oligomycin	8-10	*D. rerio*	10-20 μM	Not found
		F. heteroclitus	15-30 μM	Not found
FCCP	6-8	*D. rerio*	3-5 μM	Not found
		F. heteroclitus	2 μM	Not found
Sodium azide	25+	*D. rerio*	10 mM	Not found
		F. heteroclitus	20-30 mM	Not found
		**Skeletal Muscle**		
**Drug**	**Measurement cycles**	**Species**	**Range of concentrations tested**	**Final concentration**
Oligomycin	8-10	*D. rerio*	10 μM	Not found
FCCP	6-8	*D. rerio*	2-5 μM	Not found
Sodium azide	25+	*D. rerio*	10 mM	Not found

Note: * Measurement cycle consisted of 1 min mix, 1 min wait, 2 min measure
