# Supplementary material for: High-Throughput Tissue Bioenergetics Analysis Reveals Identical Metabolic Allometric Scaling for Teleost Hearts and Whole Organisms
Source: PLoS One. 2015 Sep 14;10(9):e0137710. doi: 10.1371/journal.pone.0137710 (PMC4569437; doi:10.1371/journal.pone.0137710)
Supplement: S3 Table — Slopes and Y intercepts for log oxygen consumption rates of whole organism, hearts and brains plotted against log body mass for all five species combined and for each individual species. (PDF) [file pone.0137710.s006.pdf]

S3 Table. Detailed statistics on regression analyses. Slopes and Y intercepts for log oxygen consumption rates of whole organism, hearts and brains plotted against log body mass for all five species combined and for each individual species.

	**All five species**			***Danio rerio***			***Fundulus heteroclitus***			***Gambusia holbrooki***			***Oryzias latipes***			***Pimephales promelas***		
	Org.[b]	Heart	Brain	Org.	Heart	Brain	Org.	Heart	Brain	Org.	Heart	Brain	Org.	Heart	Brain	Org.	Heart	Brain
Slope (*b*)	**0.5218**	**0.5504**	**-0.0886**	**0.5968**	**0.5877**	**-0.0277**	**0.7792**	**0.7084**	**-0.1607**	**0.5268**	**0.5033**	**0.08341**	**0.4099**	**0.3713**	**0.01938**	**1.072**	**1.040**	**0.1320**
Y Intercept	-0.4596	-3.079	-2.962	-0.4549	-2.901	-2.920	-0.4105	-3.130	-3.035	-0.4790	-3.241	-2.858	-0.4714	-3.272	-2.874	-0.2666	-2.919	-2.849
**Std. Error**																		
Slope	0.05091	0.06466	0.03831	0.1006	0.08579	0.04394	0.1185	0.1774	0.1592	0.1745	0.1977	0.06331	0.1725	0.1282	0.04877	0.1104	0.1483	0.07492
Y Intercept	0.02454	0.03181	0.01876	0.04986	0.04306	0.02234	0.03807	0.05699	0.05113	0.07211	0.08019	0.02479	0.1157	0.08710	0.03297	0.03963	0.05323	0.02744
**95% Confidence Intervals**																		
Slope	0.4220 to 0.6216	0.4236 to 0.6771	-0.1637 to - 0.01353	0.3939 to 0.7996	0.4149 to 0.7606	-0.1164 to 0.06087	-0.4888 to -0.3322	-3.247 to -3.013	-3.141 to -2.930	0.1659 to 0.8878	0.09321 to 0.9133	-0.0482 to 0.2151	0.05466 to 0.7652	0.1087 to 0.6339	-0.0806 to 0.1195	0.8256 to 1.318	0.7097 to 1.371	-0.0407 to 0.3048
Y Intercept	-0.5077 to -0.4116	-3.141 to -3.016	-2.999 to -2.925	-0.5554 to -0.3544	-2.988 to -2.814	-2.96 to -2.87	0.5356 to 1.0230	0.3436 to 1.073	-0.4880 to 0.1665	-0.6282 to -0.329	-3.408 to -3.075	-2.909 to -2.806	-0.7098 to -0.2330	-3.450 to -3.093	-2.941 to -2.806	-0.3549 to -0.1783	-3.038 to -2.801	-2.912 to -2.785
**Goodness of Fit**																		
R^2	0.4358	0.3426	0.03786	0.4387	0.5050	0.00921	0.6245	0.3801	0.03775	0.2839	0.2275	0.07635	0.1843	0.2305	0.00588	0.9040	0.8310	0.2796
**Sample size[a]**																		
Analyzed	138	141	138	47	48	45	28	28	28	25	24	23	27	30	29	12	12	10
Outliers	5	1	1	1	0	3	0	0	0	0	1	2	3	0	1	0	0	0

Note: [a]Total number of fish tested is obtained by combining number of samples in analyzed and outliers rows. [b]Org. -Whole organism.
