# Supplementary material for: High-Throughput Tissue Bioenergetics Analysis Reveals Identical Metabolic Allometric Scaling for Teleost Hearts and Whole Organisms
Source: PLoS One. 2015 Sep 14;10(9):e0137710. doi: 10.1371/journal.pone.0137710 (PMC4569437; doi:10.1371/journal.pone.0137710)
Supplement: S4 Table — Comparison of exponent b between log of heart and brain oxygen consumption rates (OCR) with log of whole organismal metabolic rates (RMO2). P and F values are calculated by testing if the slope of the curve log of tissue OCR:RMO2 plotted against log body mass is equal to zero. (PDF) [file pone.0137710.s007.pdf]

S4 Table. Heart and brain scaling exponent comparison. Comparison of exponent *b* between log of heart and brain oxygen consumption rates (OCR) with log of whole organismal metabolic rates (RMO_2). *P* and *F* values are calculated by testing if the slope of the curve log of tissue OCR:RMO_2 plotted against log body mass is equal to zero.

**Hearts vs Whole organism**	**All five species**	***Danio rerio***	***Fundulus heteroclitus***	***Gambusia holbrooki***	***Oryzias latipes***	***Pimephales promelas***
P value	0.1424	0.4215	0.7667	0.2155	0.3853	0.0511
**F *(DFn, DFd)***	2.176 *(1,141)*	0.6577 *(1,46)*	0.08992 *(1,26)*	1.6222 *(1,23)*	0.7779 *(1,28)*	4.909 *(1,10)*
**Brains vs Whole organism**						
P value	<0.0001	<0.0001	<0.0001	0.1502	0.0204	<0.0001
**F *(DFn, DFd)***	47.27 *(1,139)*	27.07 *(1,46)*	23.42 *(1,26)*	2.216 (*(1,23)*	6.048 *(1,28)*	45.76 (*1,8)*
