# Supplementary material for: High-Throughput Tissue Bioenergetics Analysis Reveals Identical Metabolic Allometric Scaling for Teleost Hearts and Whole Organisms
Source: PLoS One. 2015 Sep 14;10(9):e0137710. doi: 10.1371/journal.pone.0137710 (PMC4569437; doi:10.1371/journal.pone.0137710)
Supplement: S5 Table — Slopes and Y intercepts for log oxygen consumption rates of hearts plotted against log heart mass for all five species combined and for Danio rerio and Fundulus heteroclitus. (PDF) [file pone.0137710.s008.pdf]

S5 Table. Detailed statistics for heart OCR versus heart mass. Slopes and Y intercepts for log oxygen consumption rates of hearts plotted against log heart mass for all five species combined and for *Danio rerio and Fundulus heteroclitus.*

	**All five species**	***Danio rerio***	***Fundulus heteroclitus***
Slope (*b*)	0.5196	0.4722	0.6588
Y Intercept	-1.675	-1.709	-1.210
**Std. Error**			
Slope	0.06771	0.06381	0.1781
Y Intercept	0.2161	0.1976	0.5718
**95% Confidence Intervals**			
Slope	0.3869 to 0.6523	0.3437 to 0.6008	0.2926 to 1.025
Y Intercept	-2.099 to -1.252	-2.107 to -1.311	-2.385 to -0.034
**Goodness of Fit**			
R^2	0.3151	0.5435	0.3448
**Sample size[a]**			
Analyzed	130	48	28
Outliers	0	0	0

Note: [a]Total number of fish tested is obtained by combining number of samples in analyzed and outliers rows.
